# Supplementary material for: Activation mechanism of the class D fungal GPCR dimer Ste2
Source: Nature. 2022 Mar 16;603(7902):743–8. doi: 10.1038/s41586-022-04498-3 (PMC8942848; doi:10.1038/s41586-022-04498-3)
Supplement: Supplementary file 1 — Reporting Summary [file 41586_2022_4498_MOESM1_ESM.pdf]

nature research

Corresponding author(s): Christopher G. Tate

Last updated by author(s): Nov 29, 2021

Reporting Summary

Nature Research wishes to improve the reproducibility of the work that we publish. This form provides structure for consistency and transparency in reporting. For further information on Nature Research policies, see our Editorial Policies and the Editorial Policy Checklist.

Statistics

For all statistical analyses, confirm that the following items are present in the figure legend, table legend, main text, or Methods section.

n/a	Confirmed	
☒	☐	The exact sample size (*n*) for each experimental group/condition, given as a discrete number and unit of measurement
☒	☐	A statement on whether measurements were taken from distinct samples or whether the same sample was measured repeatedly
☒	☐	The statistical test(s) used AND whether they are one- or two-sided<br>*Only common tests should be described solely by name; describe more complex techniques in the Methods section.*
☒	☐	A description of all covariates tested
☒	☐	A description of any assumptions or corrections, such as tests of normality and adjustment for multiple comparisons
☐	☒	A full description of the statistical parameters including central tendency (e.g. means) or other basic estimates (e.g. regression coefficient) AND variation (e.g. standard deviation) or associated estimates of uncertainty (e.g. confidence intervals)
☒	☐	For null hypothesis testing, the test statistic (e.g. *F*, *t*, *r*) with confidence intervals, effect sizes, degrees of freedom and *P* value noted<br>*Give P values as exact values whenever suitable.*
☒	☐	For Bayesian analysis, information on the choice of priors and Markov chain Monte Carlo settings
☒	☐	For hierarchical and complex designs, identification of the appropriate level for tests and full reporting of outcomes
☐	☒	Estimates of effect sizes (e.g. Cohen's *d*, Pearson's *r*), indicating how they were calculated

Our web collection on statistics for biologists contains articles on many of the points above.

Software and code

Policy information about availability of computer code

Data collection	EPU 2.3.0.79
Data analysis	AceDRG (via CCP-EM)<br>Allosteer v1.0<br>CCP-EM v1.3<br>CHARMM36-mar2019<br>CHARMM-GUI 3.0<br>Chimera 1.13.1<br>ChimeraX v1.2.5<br>Coot 0.9-pre EL<br>cryoSPARC v3.1<br>CTFFIND 4.1 (via RELION)<br>DeepEMHancer v1.0<br>eLBOW (via PHENIX)<br>g-cluster (via GROMACS)<br>get_contact (version 2021)<br>Gctf 1.18<br>GROMACS 2019.3<br>HOLLOW v1.3<br>LINCS (no version number defined)<br>Maestro protein-preparation wizard (Schrödinger Release 2021-2)<br>Molprobity (via PHENIX)

Motioncor2 1.2.1
PHENIX 1.19.2-4158-000
phenix.resolve_cryo_em
Pymol 2.2.2
RELION 3.1
Warp 1.0.6

For manuscripts utilizing custom algorithms or software that are central to the research but not yet described in published literature, software must be made available to editors and reviewers. We strongly encourage code deposition in a community repository (e.g. GitHub). See the Nature Research guidelines for submitting code & software for further information.

Data

Policy information about availability of data

All manuscripts must include a data availability statement. This statement should provide the following information, where applicable:

- Accession codes, unique identifiers, or web links for publicly available datasets
- A list of figures that have associated raw data
- A description of any restrictions on data availability

Structures have been deposited in the Protein Data Bank (PDB; https://www.rcsb.org/), and the associated cryo-EM data has been deposited in the Electron Microscopy Data Bank (EMD; https://www.ebi.ac.uk/pdbe/emdb/) and the Electron Microscopy Public Image Archive (EMPIAR; https://www.ebi.ac.uk/empiar/): Ste2•LF (PDB 7QB9, EMD-13882, EMPIAR-10878); Ste2•Ant (PDB 7QA8, EMD-13880, EMPIAR-10877); Ste2IL•ag (PDB 7QBC, EMD-13886, EMPIAR-10879); Ste2AL•ag (PDB 7QBI, EMD-13887, EMPIAR-10879). There are no restrictions on data availability.

Field-specific reporting

Please select the one below that is the best fit for your research. If you are not sure, read the appropriate sections before making your selection.

[x] Life sciences   [ ] Behavioural & social sciences   [ ] Ecological, evolutionary & environmental sciences

For a reference copy of the document with all sections, see nature.com/documents/nr-reporting-summary-flat.pdf

Life sciences study design

All studies must disclose on these points even when the disclosure is negative.

Sample size	Sample size was not predetermined. For the cryo-EM structure, the sample size used was sufficient because it produced a structure at sufficient resolution to identify side chains. For SEC and SDS-PAGE gels, experiments were performed twice or thrice as indicated and as there was no major differences between the experiments, the sample size was deemed sufficient.
Data exclusions	During the generation of the 3D cryo-EM structure, particles that did not align well with the major population (i.e. they were damaged, poor signal to noise, different conformation, lacking a subunit) were excluded from the data set. Inclusion of 'bad' particles would have had a detrimental effect on the overall resolution of the structure. No exclusions were made in biochemical assays.
Replication	Structure determination was performed once. Replication was not required because each structure represents the average structure of ca. 130,000 molecules for Ste2•Ant, 68,000 molecules for Ste2•LF, and ca. 65,000 molecules for Ste2IL•Ag and Ste2AL•Ag from data collections performed on different days with different input materials. A number of independent experiments were performed for biochemical assays and purifications as reported in the manuscript, and all attempts at replication were successful.
Randomization	This study did not allocate experimental groups thus no randomisation was required for the reported experiments. All variables could be well controlled.
Blinding	Experimental results were all quantitative and did not require group allocation or any subjective analysis, thus no experiments were performed with blinding

Reporting for specific materials, systems and methods

We require information from authors about some types of materials, experimental systems and methods used in many studies. Here, indicate whether each material, system or method listed is relevant to your study. If you are not sure if a list item applies to your research, read the appropriate section before selecting a response.

Materials & experimental systems

n/a	Involved in the study	
☒	☐	Antibodies
☐	☒	Eukaryotic cell lines
☒	☐	Palaeontology and archaeology
☒	☐	Animals and other organisms
☒	☐	Human research participants
☒	☐	Clinical data
☒	☐	Dual use research of concern

Methods

n/a	Involved in the study	
☒	☐	ChIP-seq
☒	☐	Flow cytometry
☒	☐	MRI-based neuroimaging

Eukaryotic cell lines

Policy information about cell lines

Cell line source(s)	Trichoplusia ni (Expressions Systems & Thermo Fisher)
Authentication	The cell lines were not authenticated by the authors. The supplier maintained the cell line and did not specify means of authentication.
Mycoplasma contamination	The cell lines were not tested by the authors for mycoplasma contamination as this was performed by the supplier.
Commonly misidentified lines (See ICLAC register)	No commonly misidentified cell lines were used
